# Ginger and Propolis Exert Neuroprotective Effects against Monosodium Glutamate-Induced Neurotoxicity in Rats

**DOI:** 10.3390/molecules22111928

**Published:** 2017-11-08

**Authors:** Usama K. Hussein, Nour El-Houda Y. Hassan, Manal E.A. Elhalwagy, Amr R. Zaki, Huda O. Abubakr, Kalyan C. Nagulapalli Venkata, Kyu Yun Jang, Anupam Bishayee

**Affiliations:** 1 Department of Zoology, Faculty of Science, Beni-Suef University, Beni Suef 62511, Egypt; usamalithy@science.bsu.edu.eg; 2 Department of Pathology, Chonbuk National University Medical School, Research Institute of Clinical Medicine of Chonbuk National University-Biomedical Research Institute of Chonbuk National University, Hospital and Research Institute for Endocrine Sciences, Jeonju 54896, Korea; 3 Department of Toxicology and Forensic Medicine, Faculty of Veterinary Medicine, Beni-Suef University, Beni Suef 62511, Egypt; nourelhoudayassein@yahoo.com; 4 Faculty of Science, Al Faisaliah Campus, King Abdulaziz University, Jeddah 21453, Saudi Arabia; manalelhalwagy2003@yahoo.com; 5 Department of Forensic Medicine and Clinical Toxicology, Faculty of Medicine, Beni-Suef University, Beni Suef 62511, Egypt; amrzaki2030@yahoo.com; 6 Department of Biochemistry and Chemistry of Nutrition, Faculty of Veterinary Medicine, Cairo University, Giza 12613, Egypt; huda.omar@cu.edu.eg; 7 Department of Pharmaceutical Sciences, College of Pharmacy, Larkin University, Miami, FL 33169, USA; kvenkata@ULarkin.org

**Keywords:** Monosodium glutamate, ginger, propolis, oxidative stress, β-amyloid, neurotoxicity

## Abstract

Central nervous system cytotoxicity is linked to neurodegenerative disorders. The objective of the study was to investigate whether monosodium glutamate (MSG) neurotoxicity can be reversed by natural products, such as ginger or propolis, in male rats. Four different groups of Wistar rats were utilized in the study. Group A served as a normal control, whereas group B was orally administered with MSG (100 mg/kg body weight, via oral gavage). Two additional groups, C and D, were given MSG as group B along with oral dose (500 mg/kg body weight) of either ginger or propolis (600 mg/kg body weight) once a day for two months. At the end, the rats were sacrificed, and the brain tissue was excised and levels of neurotransmitters, ß-amyloid, and DNA oxidative marker 8-OHdG were estimated in the brain homogenates. Further, formalin-fixed and paraffin-embedded brain sections were used for histopathological evaluation. The results showed that MSG increased lipid peroxidation, nitric oxide, neurotransmitters, and 8-OHdG as well as registered an accumulation of ß-amyloid peptides compared to normal control rats. Moreover, significant depletions of glutathione, superoxide dismutase, and catalase as well as histopathological alterations in the brain tissue of MSG-treated rats were noticed in comparison with the normal control. In contrast, treatment with ginger greatly attenuated the neurotoxic effects of MSG through suppression of 8-OHdG and β-amyloid accumulation as well as alteration of neurotransmitter levels. Further improvements were also noticed based on histological alterations and reduction of neurodegeneration in the brain tissue. A modest inhibition of the neurodegenerative markers was observed by propolis. The study clearly indicates a neuroprotective effect of ginger and propolis against MSG-induced neurodegenerative disorders and these beneficial effects could be attributed to the polyphenolic compounds present in these natural products.

## 1. Introduction

Monosodium glutamate (MSG, C_5_H_8_NO_4_Na) is the sodium salt of glutamic acid. Encoded E621, it is a food additive from a group of flavor enhancers, used in a wide range of foods, such as soups, sauces, mixed condiments, chips, meat products, and puddings. MSG is a natural neurotransmitter in the brain [1]. In humans, the daily intake of free glutamate is greater than 1 g in different forms and the average daily intake of total glutamate is 10 g [2]. High daily intake of MSG results in accumulation and rise of glutamic acid in the blood [3]. The protein-bound glutamate become free only after it goes to the small intestine, hence protein-bound glutamate has no such effect of enhancing the taste of food at this level. Recent evidence suggests that the taste and palatability of MSG are mediated through specific glutamate receptors present in the stomach and intestine [4]. Several studies highlighted the deleterious effects of MSG on many organs when consumed daily [1,5,6,7,8,9].

Glutamic acid is a non-essential amino acid that primarily serves as an important excitatory neurotransmitter in the central nervous system (CNS), but also serves as an energy source for certain tissues and a substrate for glutathione synthesis [10]. Free glutamic acid can cause problems because brain tissues have many receptors for it and some areas, such as the hypothalamus, do not have an impermeable blood–brain barrier. Thus, free glutamic acid from food sources can get into the brain, injuring and frequently killing neurons, and many allergic reactions have also been reported. All commercially produced glutamic acid in food manufacturing and chemical plants is termed “MSG”. When consumed in large quantities, it may have impacts on cell growth and chromosomes and may lead to cancer development [11,12]. Furthermore, long-term intake of MSG was shown to induce hyperphagia, obesity, asthma, memory impairment, and damage to hypothalamic neurons [13].

Propolis (bee glue) is the resinous substance collected by bees from the leaf buds and barks of trees, especially poplar and conifer trees. Propolis has a long history of use in folk medicine. It appears to have antibacterial, anti-inflammatory [14], antioxidant [15], and immune-stimulating activity [16]. Most of these effects have been related to the antioxidant and free radical scavenging properties of propolis [17]. Propolis exhibited remarkable in vitro antioxidant activity at different concentrations, which may be attributed to its flavonoid contents [18] including aldehydes, caffeic acid, and caffeic acid phenethyl ester [19,20].

Ginger (*Zingiber officinale*) has been used as a spice for over 2000 years. Its roots and the obtained extracts contain polyphenolic compounds 6-gingerol and its derivatives, which possess high antioxidant activity [21]. Phytochemical studies of ginger have shown that it possesses anti-inflammatory, antioxidant, and potential cancer preventive activity [21,22,23]. As in traditional medicine, women consume rhizomes of ginger during ailment, illness, and confinement [24], and as carminatives for relieving flatulence. Further, ginger oil has been found to be an inhibitor of cyclooxygenase and lipoxygenase enzymes [25]. However, the remarkable beneficial effects of its constituents, including shoagaols, gingerols, and phenyl-ketone derivatives, were in CNS injury [26]; the enhancement of ginger treatment should be confirmed in both in vitro and in vivo studies. Therefore, ginger and propolis could be promising natural agents for mitigating the pathological squeals of reactive oxygen species (ROS)-induced neurodegeneration.

The objective of the current study was to explore the neuroprotective effects of ginger and propolis against MSG-induced neuropathological and toxicological changes. This effect was evident by neurotransmitter and electrochemical assessment in addition to evaluating the oxidative stress markers and antioxidant activity of both treatments in vitro and in vivo in comparison to a standard antioxidant.

## 2. Results

**Figure 1:**
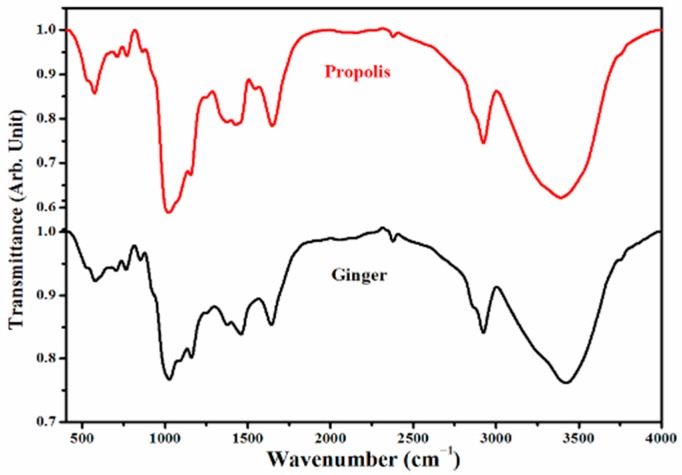
Fourier transform infrared spectra (FTIR) of ginger and propolis in the range of 400–4000 cm^−1^.

To confirm the chemical components of ginger and propolis, Fourier transform infrared (FTIR) spectra were measured for ginger and propolis particles with the same sizes; the results are depicted in Figure 1. The spectral region focused on 400 and 4000 cm^−1^, which contained more appropriate information on the chief chemical functional groups. The results showed a slight difference in the chemical group bands between ginger and propolis. Some of the characteristic chemical group bands changed in wave number and/or transmittance between both substances. Detailed peak positions and assignments of both ginger and propolis samples are listed in Table 1.

**Figure 2:**
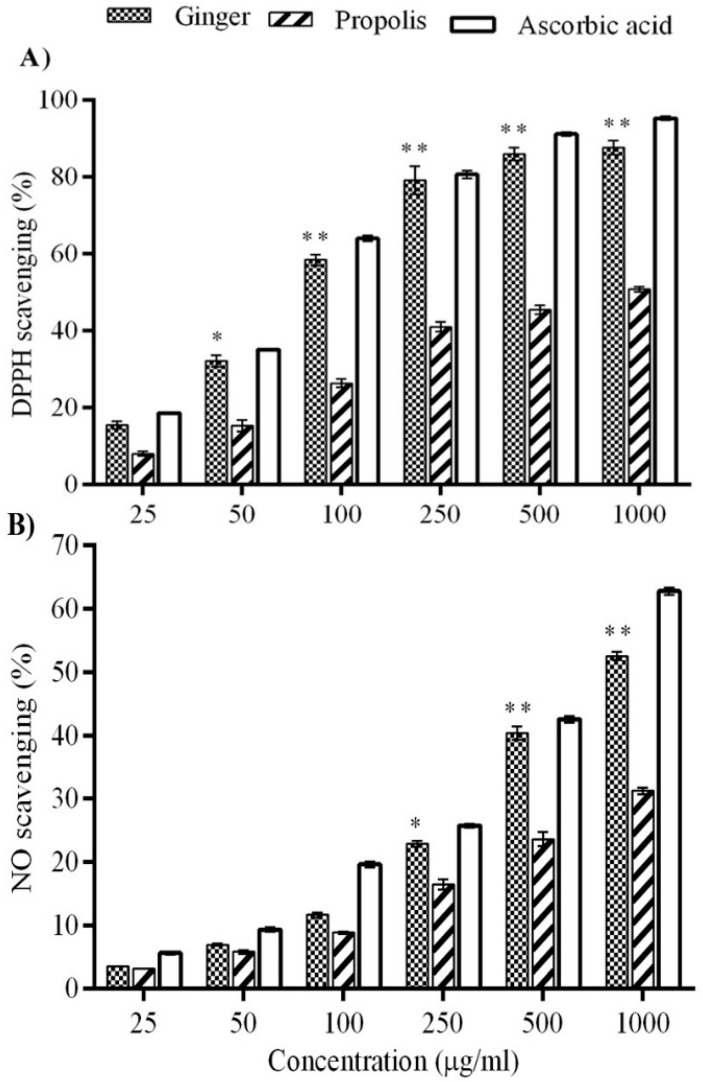
DPPH and NO scavenging activity of ginger and propolis. (**A**) DPPH scavenging activity of ginger has increased compared to propolis in a concentration-dependent manner; (**B**) the scavenging activity of ginger against NO has increased more than propolis in a concentration-dependent manner. Ascorbic acid was used as a standard flavonoid. Data are expressed as mean ± SEM (*n* = 6). * *p* < 0.01, ** *p* < 0.001 compared to propolis.

α,α-Diphenyl-β-picrylhydrazyl (DPPH) is a stable free radical with characterized absorption spectrophotometric bands. Therefore, it is considered a useful reagent for determining the free radical scavenging activities of various compounds. As shown in Figure 2A, ginger was capable of scavenging DPPH free radicals greater than propolis in a concentration-dependent manner through the activity of hydrogen donation at 50, 100, 250, 500 and 1000 mg/mL in comparison to ascorbic acid as a positive antioxidant control. Nitric oxide in Figure 2B is responsible for several pathologic and physiologic events due to its toxicity with a free radical character. The moderate NO scavenging activity of ginger and propolis in comparison to ascorbic acid is shown in Figure 2B. The nitrite production, after incubation of sodium nitroprusside solution at 25 °C for 150 min, was depleted by ginger in a concentration-dependent manner.

**Figure 3:**
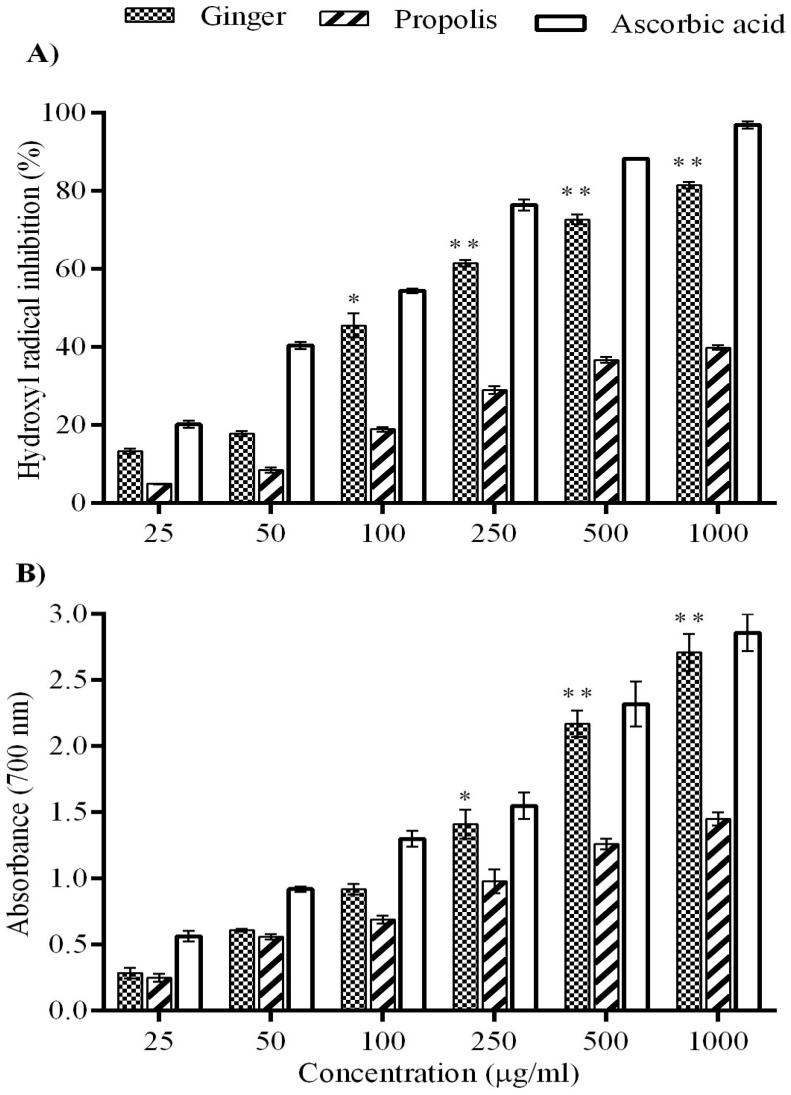
Hydroxyl radical scavenging activity and reducing power effect of ginger and propolis. (**A**) The superior inhibitory effect of ginger against hydroxyl radical in a concentration-dependent manner; (**B**) the superior reducing power effect of ginger in a concentration-dependent manner. Ascorbic acid was used as a standard flavonoid. Data are expressed as mean ± SEM (*n* = 6). * *p* < 0.01, ** *p* < 0.001.

Hydroxyl radical is a highly reactive oxygen free radical. It attacks nucleic acids, membrane fatty acids, and proteins, causing lipid peroxidic reactions. Figure 3A showed that either ginger or propolis exhibited scavenging activity against these hydroxyl radicals generated in the Fenton reaction in a concentration-dependent manner at 50, 100, 250, 500, and 1000 mg/mL, in comparison to ascorbic acid as a positive antioxidant control. The investigation of reducing power was assayed based on the transformation of Fe^3+^ to Fe^2+^ in the presence of ginger and propolis, each with the reference antioxidant compound, ascorbic acid. The total reducing ability of ginger was appreciated than propolis in a concentration dependent manner as depicted in Figure 3B.

**Figure 4:**
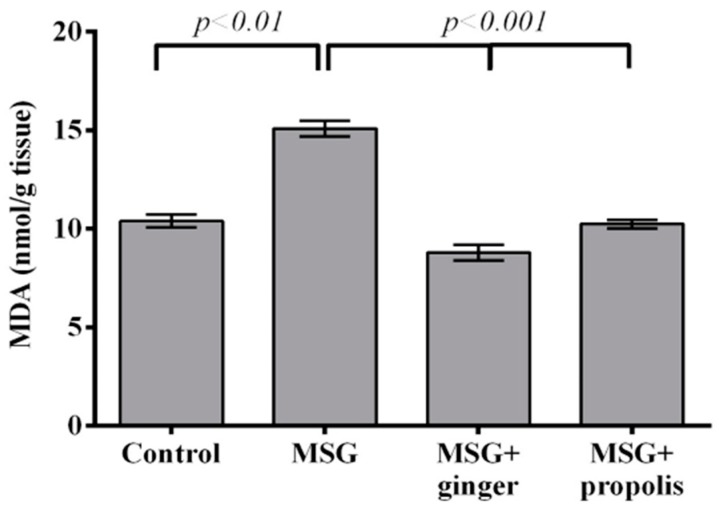
Effects of ginger and propolis aqueous extracts on the brain tissue MDA level of the experimental groups. Data are expressed as mean ± SEM (*n* = 6). Comparisons were made between normal control and MSG control group, and between MSG control and MSG plus ginger or propolis supplement groups.

**Figure 5:**
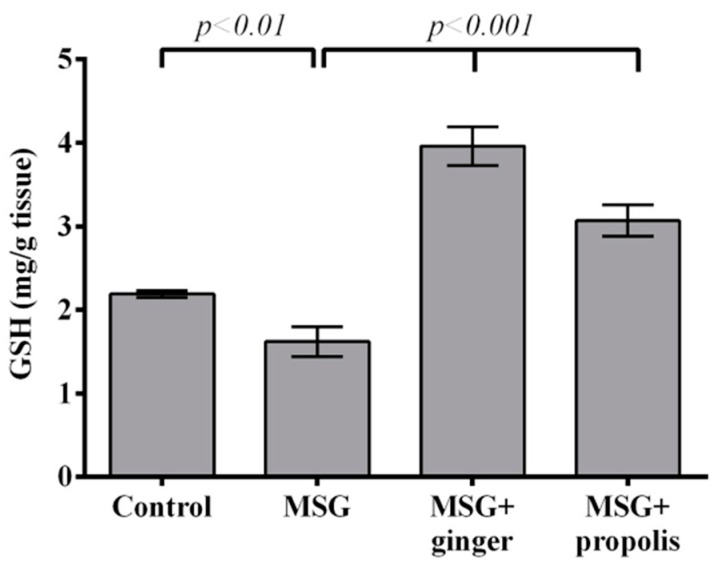
Effects of ginger and propolis aqueous extracts on brain tissue GSH level of the experimental groups. Data are expressed as mean ± SEM (*n* = 6). Comparisons were made between normal control and MSG control group, and also between MSG control and MSG plus ginger or propolis supplement groups.

**Figure 6:**
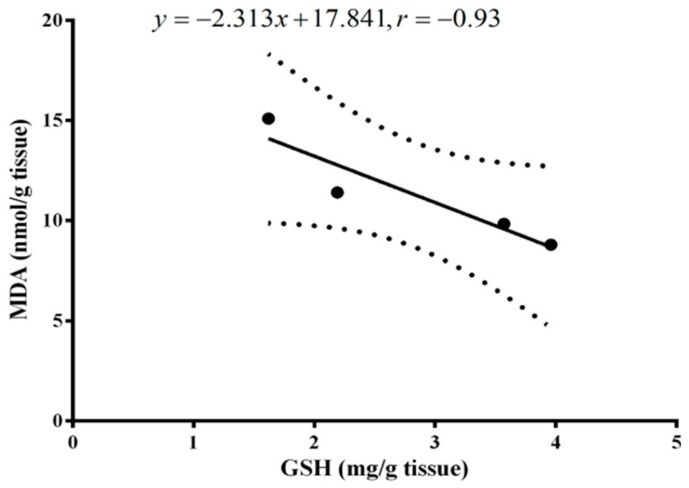
Relationship between brain GSH and MDA levels two months following MSG administration in the experimental groups. The correlation coefficient is −0.93, i.e., an inverse relationship between the GSH and MDA after two months of MSG administration. The distance between the dotted curves is the correlation area, which involves the data, and the line represents the regression strength indicator, i.e., the closer the data to linear line, the stronger the correlation.

The lipid peroxidation level in the brain tissue after MSG exposure in the presence or absence of ginger or propolis treatment is presented in Figure 4. The lipid peroxidation marker MDA increased significantly (*p* < 0.01) in the brain tissue of MSG-treated rats compared to the normal control. A significant recovery (*p* < 0.001) was observed in rats administered ginger along with MSG and recovered with propolis, but with less efficiency than ginger. The brain GSH level significantly declined in MSG-treated rats two months post-treatment compared to the normal control. On the other hand, administration of ginger restored and significantly enhanced the GSH level (*p* < 0.001) at two months greater than propolis (Figure 5). The present study showed that a declining GSH level after two months of MSG administration was closely correlated (r = −0.93) with the increased MDA level (Figure 6).

**Figure 7:**
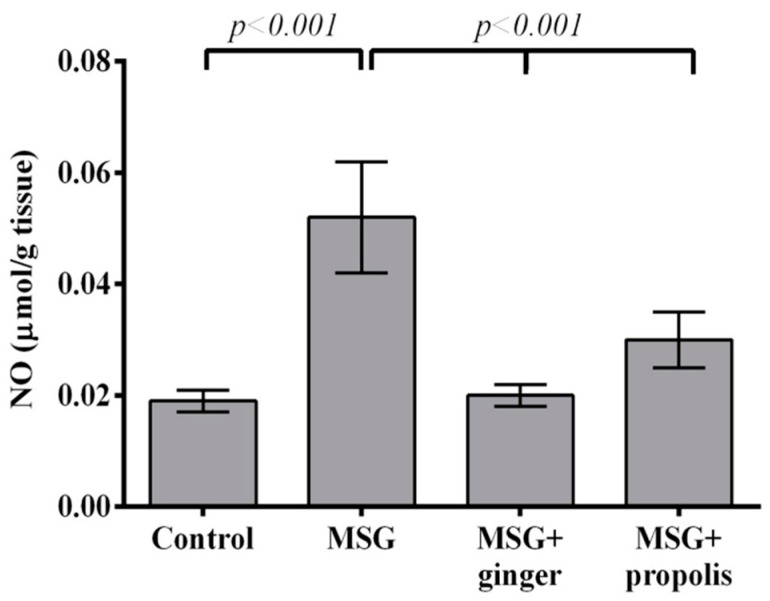
Effects of aqueous extract of ginger and propolis on NO level in the brain of experimental groups. Data are expressed as mean ± SEM (*n* = 6). Comparisons were made between normal control and MSG control group, and also between MSG control and MSG plus ginger or propolis supplement groups.

The nitric oxide (NO) level was increased in MSG-treated rats at two months post-dose in comparison with normal control (Figure 7). On the other hand, ginger significantly (*p* < 0.001) decreased the NO level at two months post-treatment compared to MSG-treated rats and caused a modest inhibition of NO upon propolis treatment.

**Figure 8:**
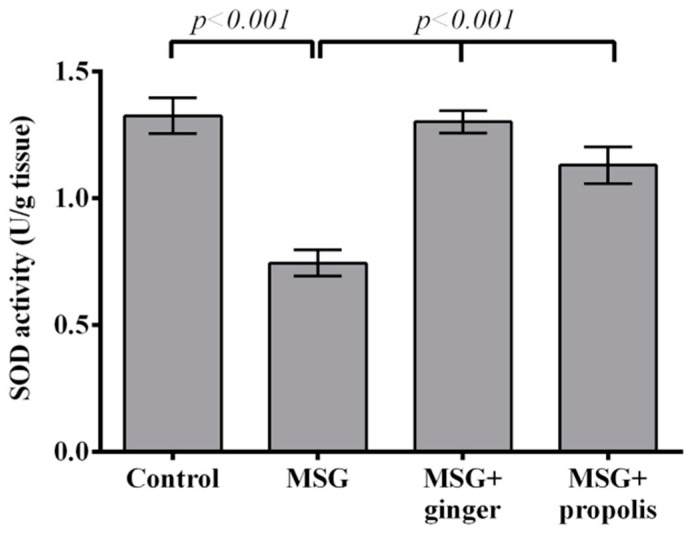
Effects of aqueous extract of ginger and propolis on SOD enzyme activity in the brain tissue of experimental groups. Data are expressed as mean ± SEM (*n* = 6). Comparisons were made between normal control and MSG control groups, and also between MSG control and MSG plus ginger or propolis supplement groups.

**Figure 9:**
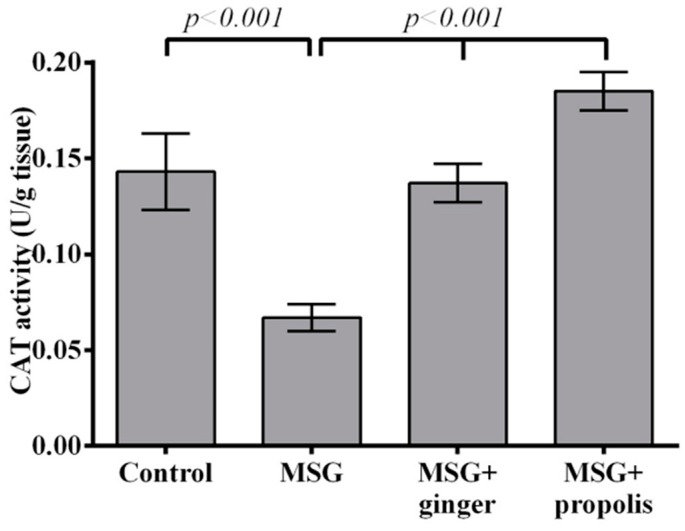
Effects of aqueous extract of ginger and propolis on Cat enzyme activity in the brain tissue of various experimental groups. Data are expressed as mean ± SEM (*n* = 6). Comparisons were made between normal control and MSG control groups, and also between MSG control and MSG plus ginger or propolis supplement groups.

**Figure 10:**
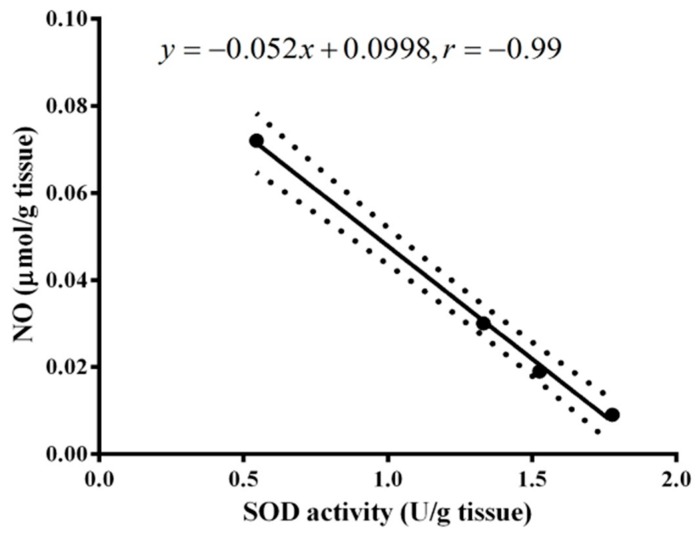
Relationship between brain NO and SOD levels two months following MSG administration of all the experimental groups. The correlation coefficient is −0.99, i.e., an inverse relationship between the NO and SOD at two months post-treatment. The distance between the dotted curves is the correlation area which involves the data and the line represents the regression strength indicator, i.e., the close the data to linear line, the strong the correlation.

Administration of MSG significantly depleted SOD and CAT activities in MSG-treated rats at two months post-treatment compared to normal control. Orally administration of ginger caused a significant (*p* < 0.001) increase in SOD at two months compared to MSG-treated rats; these findings seem to be more significant than for propolis (Figure 8). On the other hand, propolis administration improved CAT enzyme activity with greater efficiency than ginger (Figure 9). Interestingly, a strong inverse relationship (r = −0.99) was found between NO level and SOD activity in MSG-treated rats at two months post-treatment (Figure 10).

**Figure 11:**
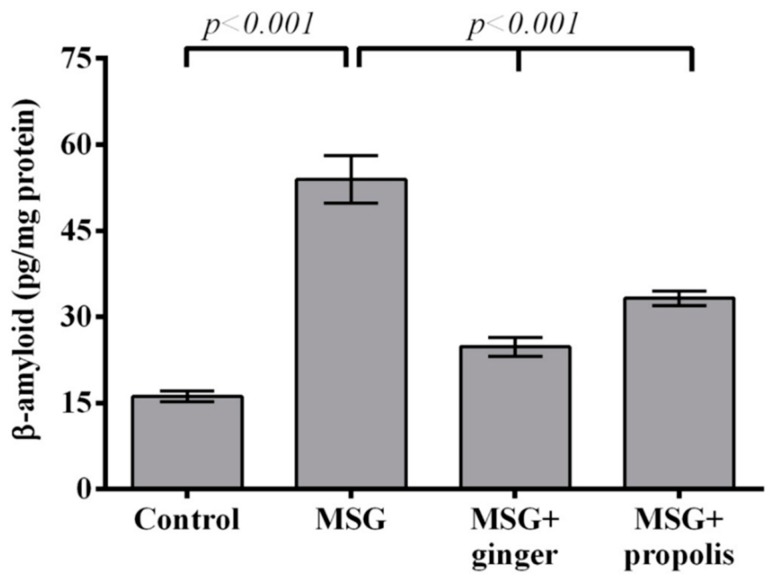
Cerebral cortex free β-amyloid peptides at two months post-treatment. Data are expressed as mean ± SEM (*n* = 6). Comparisons were made between normal control and MSG control groups, and also between MSG control and MSG plus ginger or propolis supplement groups.

Concomitant with the above results, a significant (*p* < 0.001) accumulation of free β-amyloid peptides was seen in the brain of control MSG-treated animals—more than double fold that of the control. On the contrary, administration of either ginger or propolis partially decreased to reach the normal control level. Ginger supplementation achieved a superior inhibitory effect compared to propolis (Figure 11).

**Figure 12:**
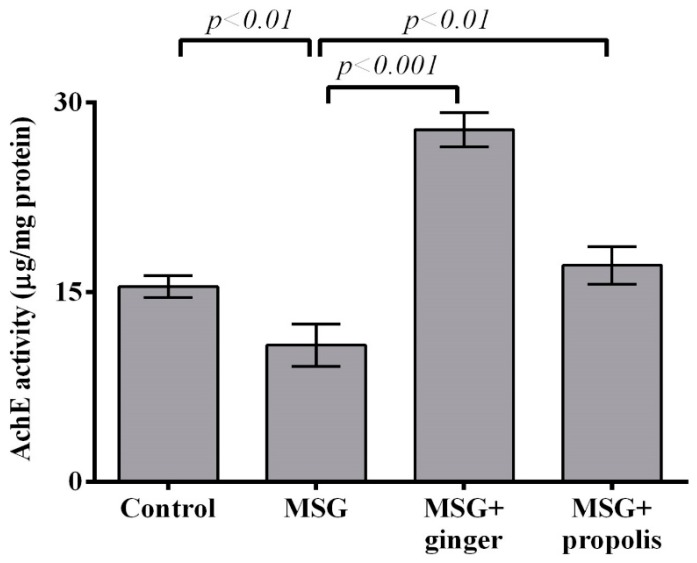
Cerebral cortex acetylcholinesterase (AchE) activity at two months post-treatment. Data are expressed as mean ± SEM (*n* = 6). Comparisons were made between normal control and MSG control groups, and also between MSG control and MSG plus ginger or propolis supplement groups.

**Figure 13:**
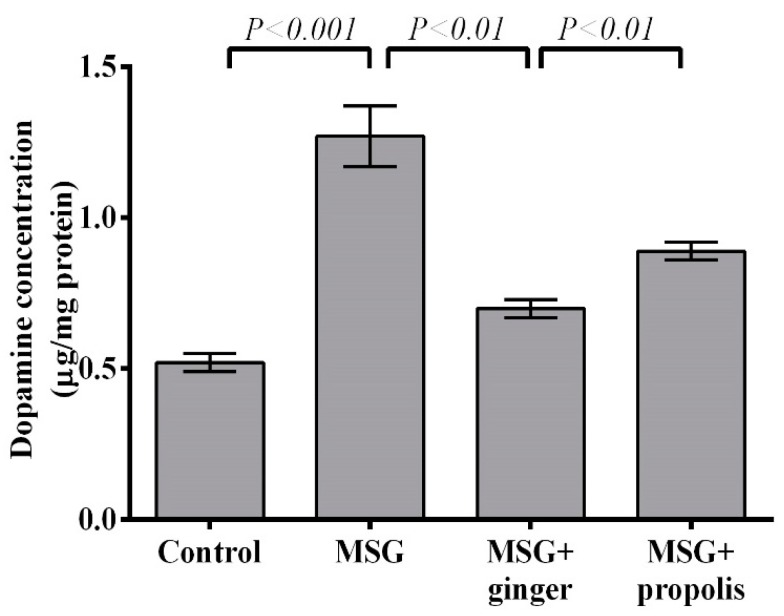
Cerebral cortex dopamine concentration at two months post-treatment. Data are expressed as mean ± SEM (*n* = 6). Comparisons were made between normal control and MSG control groups, and between MSG control and MSG plus ginger or propolis supplement groups as well.

**Figure 14:**
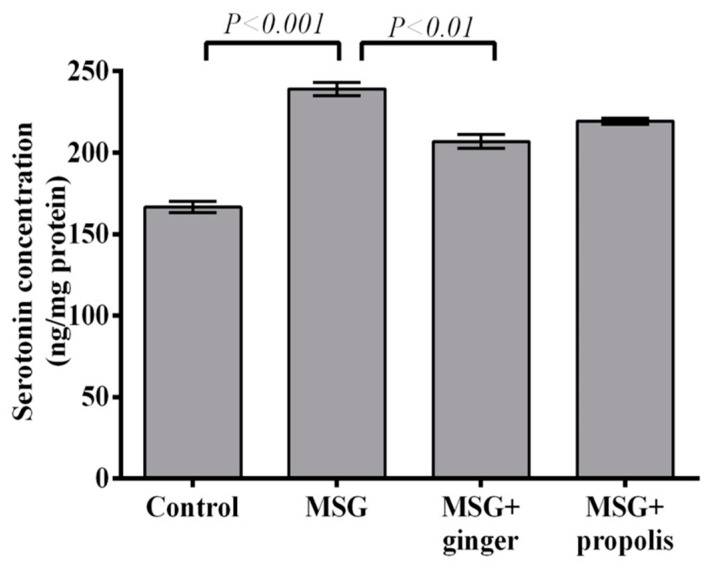
Cerebral cortex serotonin concentration at two months post-treatment. Data are expressed as mean ± SEM (*n* = 6). Comparisons were made between normal control and MSG control groups, and between MSG control and MSG plus ginger or propolis supplement groups as well.

**Figure 15:**
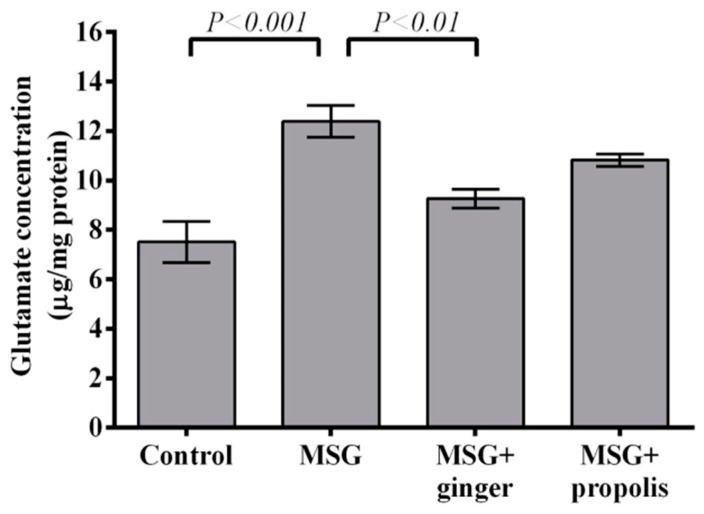
Cerebral cortex glutamate concentration at two months post-treatment. Data are expressed as mean ± SEM (*n* = 6). Comparisons were made between normal control and MSG control groups, and between MSG control and MSG plus ginger or propolis supplement groups as well. No significant difference between MSG group and MSG plus propolis as well as normal control and MSG plus ginger (data not shown).

The results depicted in Figure 12 and Figure 13 reveals that treatment with MSG induced a significant (*p* < 0.01) decrease in brain AchE activity and overexpression of dopamine neurotransmitter (*p* < 0.001) versus the control group. However, administration of ginger and propolis reversed these effects significantly versus the MSG control. The mitigating effect of ginger supplementation was found to be more pronounced than that of propolis. Moreover, the brain serotonin and glutamate neurotransmitters showed a significant (*p* < 0.001) elevation in the control MSG-treated group versus the normal control, as shown in Figure 14 and Figure 15. However, ginger supplementation restored the brain glutamate level to nearly normal. Regarding serotonin, ginger or propolis supplementation recorded a nearly equal effect on serotonin levels, despite ginger appearing more promising than propolis.

The calcium, potassium, and sodium levels in the brain homogenate of rats treated with MSG in the presence/absence of ginger or propolis are given in Table 2. The results showed a significant elevation (*p* < 0.05) of calcium and sodium levels in the control MSG-treated group versus the normal control group, concurrent with a significant reduction in potassium level (*p* < 0.05) versus the normal control. On the other hand, administration of ginger or propolis to rats significantly reduced the calcium and sodium levels. However, a significant (*p* < 0.05) elevation in the potassium level was recorded in ginger/propolis treated groups in comparison to the control MSG group.

**Figure 16:**
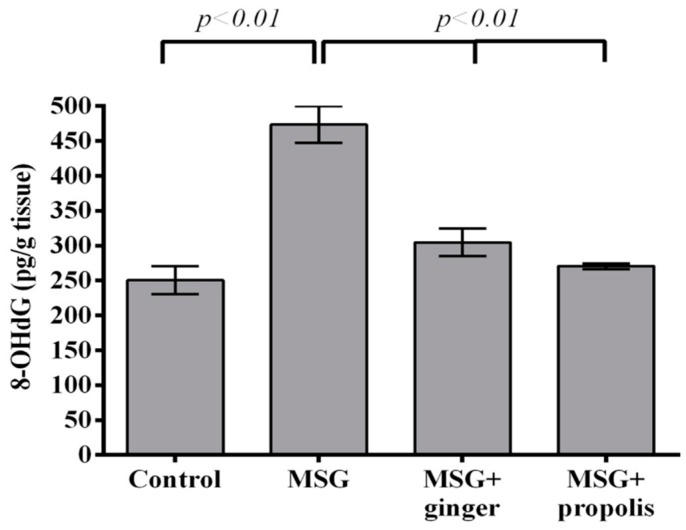
Cerebral cortex DNA 8-OHdG biomarker of oxidative stress two months after oral MSG administration with or without ginger or propolis. Data are expressed as mean ± SEM (*n* = 6). Comparisons were made between normal control and MSG control groups, and between MSG control and MSG plus ginger or propolis supplement groups as well.

8-hydroxy-2’-deoxyguanosine (8-OHdG), a biomarker of endogenous oxidative stress in the DNA of the brain, showed a pronounced and significant (*p* < 0.01) elevation in control MSG-treated rats compared to the normal control. However, the administration of ginger extract reduced this elevation in a statistically significant (*p* < 0.01) manner compared to the MSG control; propolis registered a remarkable reduction in 8-OHdG (*p* < 0.01) in comparison to the control MSG-treated group (Figure 16).

**Figure 17:**
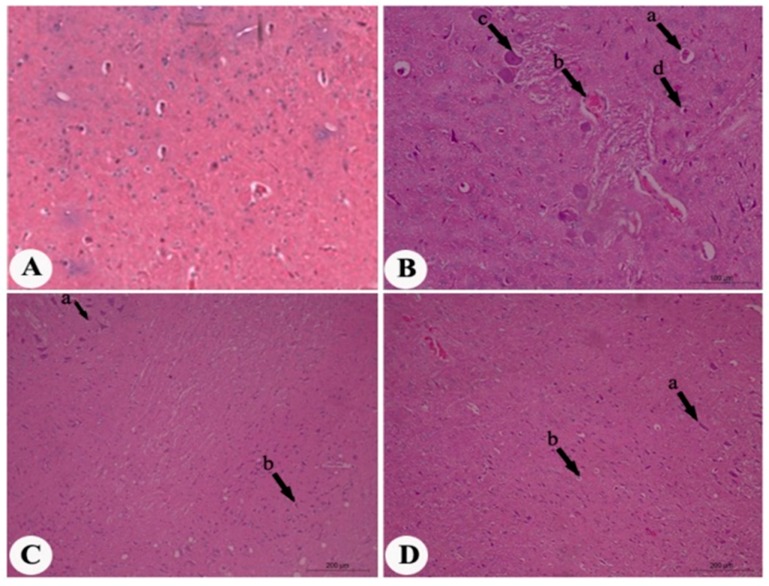
Photomicrographs of brain cerebral cortex sections stained with hematoxylin and eosin (H&E) of at two months MSG post-treatment (magnification: 400×). (**A**) Brain sample from normal control rats showing distinct neurons and normal glial cells with no vacuolation; (**B**) brain sample from rats treated with MSG showing perivascular edema (arrow a), congestion (arrow b), severe neuronal degeneration and pyknosis (arrow c), pericellular edema (arrow d); (**C**) brain samples from rats treated with MSG and ginger, showing moderate neuronal degeneration (arrow a) and mild pericellular edema (arrow b); (**D**) brain sample from rats treated with MSG and propolis, showing mild neuronal degeneration (arrow a) and mild pericellular edema (arrow b).

Figure 17A revealed distinct neurons and normal glial cells with no vacuolation in cerebral cortex specimens stained with hematoxylin and eosin (H&E) after two months in normal rats. Examination of brain tissue from rats treated with MSG showed several pathological changes, including perivascular edema, congestion, severe neuronal degeneration, pyknosis, and marked pericellular edema (Figure 17B). Regarding the samples from the ginger-treated group, moderate neuronal degeneration (arrow a) and mild pericellular edema were observed (Figure 17C), and the samples taken from the propolis-treated group showed mild neuronal degeneration and pericellular edema (Figure 17D).

## 3. Discussion

MSG is one of the most used food additives all over the world. It accumulates in the brain and causes prolonged neurotoxicity, leading to neurodegeneration and synaptic loss [3]. Glutamate salt is the excitatory neurotransmitter in the mammalian CNS, playing important roles in both physiological and pathological processes [27]. The present study demonstrated that MSG treatment elicited several toxicological consequences in our animal models. On the other hand, the potential properties of ginger and propolis aqueous extract in restoring these toxicological consequences were properly investigated. The results revealed that oxidative stress-induced brain tissues of MSG-treated rats evidenced a remarkable elevation in MDA (lipid peroxidation biomarker), 8-OHdG (marker of DNA oxidation) and nitric oxide. Mitochondrial dysfunction ultimately enhances cytokine production, which turns on genes like inducible nitric oxide synthase (iNOS), which in turn increases nitric oxide production, consistent with increased NO in our results and leading to further mitochondrial damage [9].

Concomitant to these findings, a reduction in brain total GSH, SOD, and CAT antioxidant enzymes was observed in our results, and these findings are in line with those reported previously [6,9,12,28] that describe increased oxidative stress following MSG administration in the liver and kidney of rats. GSH depletion is a positive indicator of tissue degeneration and the magnitude of depletion parallels the severity of the damage [29,30,31]. Shivasharan and colleagues [8] recorded a depletion of GSH, GST, and CAT activity, and MDA and nitrite levels in brain tissues following MSG treatment for seven days. Furthermore, DNA lesions have been identified by 8-OHdG as a biomarker of oxidative damage [32]. Oxidative damage resulting from biochemical interactions between reactive oxygen species (ROS) and target biomolecules, such as nucleic acids, lipids, and proteins, is prominently linked to the etiology and progression of numerous oncologic and neurodegenerative diseases.

On the other hand, our results revealed that ginger and propolis supplementation ameliorated these oxidative stresses and restored the antioxidant enzymes in MSG-treated rats. It has been reported that ginger has potential antioxidant properties [33], which might be attributed to the shogaols, gingerols, and other phenolic-ketone derivatives shown to be beneficial in attenuating ROS-induced CNS injury [24]. A key finding of our study was that ginger supplementation inhibited lipid peroxidation in the brain tissue, which was supported by a previous study reporting the inhibition of ascorbate/ferrous complex-induced LPO by gingerol in microsomes of the rat liver [34]. Inhibition of nitric oxide in the brain tissue beyond ginger supplementation is in line with a previous study that reported nitric oxide inhibition in activated macrophages [35]. Furthermore, 6-shogaol is one of the most bioactive components of ginger rhizome and has been shown to decrease the iNOS level [36].

The brain is particularly vulnerable to membrane lipid peroxidation due to the relatively high abundance of polyunsaturated fatty acids (PUFA), such as archidonic acid and docosohexenoic acid [37]. In this context, Butterfield and Lauderback [38] reported that an increased level of free β-amyloid peptides is an indicator of free radical propagation, which confirms our finding of elevation of free β-amyloid peptides due to MSG-induced oxidative stress. A previous study provided results consistent with our finding about the elevation of free β-amyloid peptides recorded in the brain tissues of MSG-treated rats [5], linked to harmful effects on the cognitive neurotransmitters [39]. On the other hand, these events were attenuated by ginger more efficiently than propolis supplementation, which could be attributed to the antioxidant properties in favor of ginger, as mentioned in the discussion of free radical scavenging activity in our in vitro study. Furthermore, it also might be related to its active constituent 6-gingerol, which played a key role in the attenuation of β-amyloid in another study [35].

It has been known that free radicals attack unsaturated bonds of membrane fatty acids, resulting in an autocatalytic process that can impair the function of membrane AchE [40]. The significant inhibition of AchE activity in the brains of MSG-treated rats in our findings is consistent with a previous study [41] that reported a decrease in AchE following MSG administration. Significant alterations in serotonin, dopamine, and glutamate levels in the cerebral cortex of MSG-intoxicated rats were recorded in our study. This serotonin finding was supported by a previous report that has shown changes of serotonin in cortex, hippocampus, striatum, hypothalamus, the olfactory lobe, cerebellum, and brain stem in rats exposed to MSG and aspartame [42]. On the other hand, these events were improved upon ginger and propolis administration, which might be attributed to the antioxidant properties of ginger and propolis. To our knowledge, glutamate plays a key role in the regulation of blood–brain barrier permeability [43] and the glutamate receptors in the cerebral capillaries, when overstimulated, will destabilize the blood–brain barrier. Our finding of increased glutamate level in the brain of rats exposed to MSG is in accord with a previous study [44] linking the overexpression of glutamate levels to the breakdown of the blood–brain barrier [43,45] and hippocampus and hypothalamus neurotoxicity [46]. Various investigators have previously demonstrated that ginger fractions have an anti-5HT3-receptor effect [47], which in turn contributes to fast excitatory synaptic transmission in the CNS upon its stimulation [48], and modulates the release of γ-aminobutyric acid, the exocytosis of which is enhanced by direct Ca^2+^ influx through the ionophore of presynaptic 5-HT3-receptors [49,50].

MSG may cause alterations in mitochondrial membrane lipids and antioxidant status in different parts of the brain. In this context, glutamate binding to NMDA receptors and mitochondrial dysfunction reduce ATP production, ultimately causing calcium influx [9]. The increase of Ca^+2^ and Na^+^ and decrease in K^+^ concentrations in the brain cells following MSG-excitotoxicity and overstimulation of glutamate receptors led to neuronal cell death [51,52]. Regarding the brain tissue of MSG-treated rats, Na^+^ and Ca^+2^ levels were significantly elevated and K^+^ was significantly depleted compared to the normal control group owing to MSG neurotoxicity. On the other hand, the groups treated with ginger and propolis significantly decreased in Na^+^ and Ca^+2^ concentration in the brain tissue and significantly increased in K^+^ concentration compared to the MSG-treated group. However, the activity of ginger supplementation is more pronounced than propolis, possibly due to the high antioxidant properties of ginger and its bioactive components.

DPPH is a relatively artificial free radical used to evaluate the free radical scavenging activity of various natural compounds in vitro. The scavenging ability is indicated by the degree of discoloration that is produced by the reduction of the DPPH by the H^+^ supplied by the natural compound. In this study, the results revealed that ginger and propolis extracts produced scavenging free DPPH radicals in a dose-dependent manner compared to a natural antioxidant control, ascorbic acid. The scavenging activity of DPPH by ginger was double that of propolis, suggesting that ginger displays a pronounced antioxidant capacity, which was near the levels of ascorbic acid in a dose-dependent manner. A previous report revealed that ginger extract contains a significant amount of polyphenolics, which possess strong DPPH free radical scavenging capability [53]. The reducing power of ginger and propolis was determined by the reduction of Fe^+3^ to Fe^+2^ reactions after electron donation. In our findings, ginger showed a higher reducing ability in a concentration-dependent manner than propolis, which was in line with previous studies showing the ability of phenolic compounds to reduce the power of various plant extracts [54,55]. The higher ferric reducing power of ginger might be due to the presence of an appreciable amount of polyphenols. 

A hydroxyl radical is one of the major oxygen radicals causing oxidative damage to proteins, lipids [56], and DNA [57]. The results of the present study showed a higher inhibitory potential of ginger than propolis against the OH radicals released. The mechanism may be attributed to the phenolic compounds contained in the ginger extract, as described in a previous report [53]. NO is an important free radical effector playing multiple roles in the biological system, such as neuronal messengers, antimicrobial agent, vasodilator, etc. Various studies reported its reaction with O^2^ radical to form peroxinitrite radicals (ONOO-) that cause macromolecular toxicity [58]. In the present in vitro study, ginger inhibited NO˙ radical generation remarkably, in line with ascorbic acid as a control antioxidant in a dose-dependent manner. Taken together, we can postulate that the phenolic compounds of ginger and propolis serve as antioxidants like other plant phytochemicals by acting as reducing agents that convert free radicals into stable compounds [59,60,61].

The present histopathological examination showed severe neuronal degeneration in MSG-treated rats, evidenced by perivascular edema, congestion, and pyknosis. It has been known that ROS play a role in nervous system toxicity, produced by MSG-induced oxidative stress in our findings. On the other hand, this neurotoxicity was markedly mitigated after ginger and propolis treatment, and the ameliorative effects of both treatments may be due to their antioxidant properties. Interestingly, ginger improved the histological features of the brain more than propolis, as represented by mild pericellular edema and mild neurodegeneration. Ginger is rich in polyphenolic compounds, which are characterized by the highest antioxidant values and anti-inflammatory properties [51,62].

Conclusively, the oral administration of MSG enhanced β-amyloid peptides’ accumulation and oxidative stress induction. Ginger treatment attenuated MSG-induced neurodegenerative diseases compared to the modest attenuation of propolis. These effects may interpret the conserved organic polyphenolic constituents, but these results may need further molecular investigation to justify the molecular mechanism of ginger and propolis.

## 4. Experimental Section

### 4.1. Chemicals

All chemicals and reagents were obtained from Sigma-Aldrich (St. Louis, MO, USA), Randox Chemical Company (Antrim, UK), Bio-Merieux Company (Marcy l’Etoile, France), Biodiagnostics (Giza, Egypt) and Spinreact (Girona, Spain).

### 4.2. Analyses of Ginger and Propolis Using FTIR

The propolis and ginger powder samples were prepared with potassium bromide (KBr) pellet method [63]. Infrared spectra were determined with a Perkin-Elmer Model GX FT-IR spectrophotometer at 20 °C. For each infrared spectrum, 256 interferograms was collected with a resolution of 3 cm^−1^ with 32 scans and 2 cm^−1^ intervals from the 400 to 4000 cm^−1^ region. The system was continuously purged with dry air. The reference spectra were recorded under the same conditions, but the KBr media containing no propolis/ginger powders served as the blank. Each sample spectrum was divided by the background spectrum to remove the atmospheric conditions and instrument effects to ensure the final spectrum peaks for the sample only (Thermo Nicolet User’s Guide). The resulting FTIR spectra are the average values of the relevant single spectra. The final FTIR spectra have been corrected for baseline in the region of 400–4000 cm^−1^ using Win-Pro program version 2.9 Bio-Rad (Hercules, CA, USA). Three replicated spectra were collected for every sample pressed on the attenuated total reflection (ATR) crystal.

### 4.3. Measurement of Antioxidant Activity

#### 4.3.1. DPPH-Based Free Radical Scavenging Assay

Free radical scavenging activity for the aqueous solutions of ginger and propolis was determined using a stable free radical, DPPH [64]. The assay was detected by using variable concentrations of both treatments (25–1000 µg/mL qH_2_O) and DPPH was added in the solutions (1 × 10^−4^ M in methanol). After 30 min incubation at room temperature, the level of DPPH left was determined spectrophotometrically at 517 nm. L-ascorbic acid was used as the positive antioxidant control. The activity of scavenging free radicals was calculated by the absorbance values of control according to the following equation:

DPPH radical scavenging effect (%) = [1 − (*A*_sample517nm_/*A*_control517nm_)] × 100.

#### 4.3.2. Superoxide Radical Scavenging Assay

The superoxide free radical scavenging ability of ginger and propolis fractions was detected using the method of Liu and Ng [65]. The reaction mixture, containing various concentrations of ginger and propolis solutions (25–1000 µg/mL qH_2_O), 0.1 M Tris-HCl (pH 8.0), 936 μM NADH, 300 μM nitroblue tetrazolium (NBT), and 120 μM phenazine metho-sulfate (PMS), was incubated at 25 °C for 5 min, and the absorbance was read at 560 nm spectrophotometrically. The activity of scavenging superoxide free radical was calculated by the following equation:

Superoxide scavenging activity (%) = [1 − (*A*_sample560nm_/*A*_control560nm_)] × 100.

#### 4.3.3. Nitric Oxide Radical Scavenging Activity

The scavenging activity of ginger and propolis on nitric oxide was measured according to the modified method of Sreejayan and Rao [66]. Briefly, the reaction mixture containing various concentrations of ginger and propolis (50–1000 µg/mL qH_2_O) and 5 mM sodium nitroprusside in phosphate-buffered saline (PBS) (pH 7.3) and then incubated at 25 °C for 2 h. A 2-mL aliquot of the incubated solution was diluted with 1.2 mL of Griess reagent (1% sulfanilamide in 5% phosphoric acid and 0.1% 1-naphthylethylene diamine dihydrochloride in water). The absorbance of the formed chromophore dye during diazotization of the nitrite with sulfanilamide and subsequent coupling with 1-naphthyleethylenediamine dihydrochloride was measured immediately at 560 nm L-ascorbic acid was used as the positive control. The activity of NO scavenging was calculated by the following equation:

NO scavenging activity (%) = [1 − (*A*_sample560nm_/*A*_control560nm_)] × 100.

#### 4.3.4. Ferric Reducing Potential Assay

The reducing power of the ginger and propolis was determined according to the method of Oyaizu [67]. Various concentrations of sample solutions (50–250 µg/mL qH_2_O) were mixed with 1.5 mL of 0.2 M phosphate buffer (pH 6.6) and 1.5 mL (1% *w*/*v*) potassium ferricyanide. The mixtures were incubated in water bath at 50 °C for 20 min. Later, 1.5 mL of 10% trichloroacetic acid solution (TCA) was added and the mixtures then centrifuged at 3000 rpm for 10 min. The supernatant of the mixture was mixed with 1.5 mL FeCl_3_ solution (0.1% *w*/*v*), and was measured spectrophotometrically at 700 nm. L-ascorbic acid was used as the positive control.

### 4.4. Animals and Maintenance

Pathogen-free male albino rats, initially weighing 100–130 g, were obtained from the Vacsera Research Centre (Dokki, Giza, Egypt). The experimental animals were housed in a conventional animal facility. The rats were acclimatized and accommodated to standard conditions (temperature 23 ± 1 °C, relative humidity 55 ± 5% and a 12-h photoperiod), and were kept in polycarbonated cages (6–8 rats per cage) for one week before the commencement of the experiment. The animals were kept under observation for this period before initiating the experiment to exclude any infectious animals. During the entire period of study, the rats were provided with normal basal diet (El-Nasr, Giza, Egypt) and water ad libitum. The animal procedures were conducted according to the Canadian Committee for Animal Use and Care [68], and were approved by the Zoology Department, Faculty of Science, Beni-Suef University.

### 4.5. Experimental Design for Chemoprotective Study

**Figure 18:**
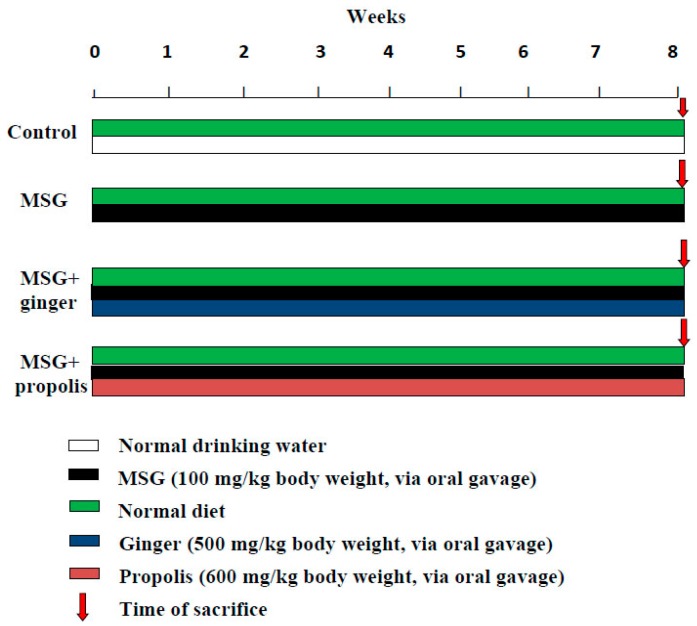
Schematic representation of experimental regimen.

Pathogen-free rats were randomly divided into four experimental groups (6–8 rats in each). The first group of rats (normal control) was provided with a normal basal diet and drinking water (Figure 18). The second group of rats was designated the toxic control and received a single daily dose of MSG (100 mg/kg body weight) by oral gavage, continued thereafter for two consecutive months based on previous studies, where they used 2 g/kg body weight [5,69,70]. The third and fourth group rats treated exactly as second group, and additionally co-treated with oral dose (500 mg/kg body weight, orally) of ginger or propolis (600 mg/kg body weight, orally) once a day by oral gavage, respectively, throughout the entire period of study. Food and water intake as well as behavioral changes were monitored every time, and the body weights of rats were recorded every week. For the last two days, MSG was discontinued, and the rats did not receive the ginger or propolis fractions. The rats were also fasted overnight prior to being sacrificed. At the end of the study, all the animals were anesthetized with diethyl ether for scarification and immobilized in a stereotactic apparatus. The scarification process was initiated by the puncture of the jugular vein to collect blood sera for measuring the various endpoint biomarkers. Moreover, the skin of the head was incised, and the frontal bone was trepanned 3 mm laterally from the bregma and another 3 mm anteriorly to the coronal suture and the dura mater and subarachnoid mater were incised. The cerebral cortex was hemisected using a sharp scalpel.

### 4.6. Brain Tissue Sampling for Biochemical Assays

At the end of the experiment, six rats from each group were sacrificed under light ether anesthesia following overnight fasting. Brain tissue samples were excised and stored at −20 °C for subsequent analysis.

#### 4.6.1. Tissue Processing Methods

Following sacrifice, the cerebral cortex was excised immediately, and divided into two parts longitudinally. One part was kept in 10% neutral-buffered formalin for histopathological studies at the histopathology unit, National Cancer Institute, Cairo, Egypt. The other part was rinsed with saline (0.9% NaCl), dried, and homogenized in a phosphate buffer (10 mM, pH 7.4) that contained 1.15% potassium chloride and 1.15% ethylene-diamine tetra-acetic acid (EDTA), and centrifuged at 3000 rpm for 15 min to obtain tissue lysates. The tissue lysates were collected and kept at −20 °C to be used for the estimation of several endpoint parameters, as described below.

#### 4.6.2. Lipid Peroxidation

An established method [71] was used to assess the level of lipid peroxides (LPO) by measuring the MDA formed in the brain tissue according to the instructions provided by the manufacturer (Biodiagnostic, Giza, Egypt). Briefly, 200 µL aliquots of lysate was added to 1 mL of thiobarbituric acid (TBA) with stabilizer and detergent and heated in a water bath at 95 °C for 30 min to form a TBA-reactive product in an acid medium. The amount of MDA formed was assayed by measuring the optical density of the supernatant at 532 nm against the standard. The results were expressed as nmol MDA formed/g tissue.

#### 4.6.3. GSH

The GSH level was measured according to the manufacturer’s protocol [72] (Spinreact). Briefly, 500 µL of the lysate were mixed with 500 µL of TCA and allowed to stand for 5 min at room temperature. After centrifugation at 3000 rpm for 15 min, 500 µL of the supernatant was mixed with 100 µL of 5,5-dithio-bis (2-nitrobenzoic acid) (DTNB) and the absorbance was measured after 25 min at 405–414 nm against the blank.

#### 4.6.4. NO

NO was measured by the method of Montgomery and Dymock [73]. NO is relatively unstable in the presence of molecular oxygen. With a short half-life of 3–5 s, it is rapidly oxidized to nitrate and nitrite, which are designated as NOx. The method depends on a Griess reaction that converts nitrite into a purple azo dye compound, which measured photometrically at 550 nm due to this chromophore compound. A high correlation between endogenous NO production and nitrate/nitrite (NOx) activities has been established.

#### 4.6.5. SOD

The SOD activity in the brain lysate was assayed by the technique of Marklund and Marklund [74], based on the rapid auto-oxidation of pyrogallol in an aqueous solution. To 1 mL of the lysate, 100 µL Tris buffer were added followed by 5 µL of pyrogallol. The change in absorbance at 430 nm was determined by subtracting the initial absorbance immediately measured after the addition of pyrogallol from the final absorbance measured after 10 min incubation.

#### 4.6.6. CAT

The CAT activity was assayed based on the manufacturer’s protocol (Randox), previously described by Fossati et al. [75]. The catalase reacts with a known quantity of H_2_O_2_; the reaction is stopped by a catalase inhibitor after exactly 1 min and the remaining H_2_O_2_ reacts with 3,5-dichloro-2-hydroxybenzene sulfonic acid in the presence of peroxidase enzyme to form a chromophore with a color intensity inversely proportional to the amount of catalase. To the aliquot of the lysate, hydrogen peroxide was added to a final concentration of 500 mM and 50 µL samples were incubated for 1 min at 25 °C before adding 200 µL of chromogen inhibitor and 500 µL of peroxidase enzyme. The mixture was incubated for 10 min at 37 °C and the absorbance was read at 500–520 nm against a blank.

#### 4.6.7. Assessment of β-Amyloid Peptides

Cerebral cortex of the brain was excised from various experimental groups and homogenized in a buffer containing 30 mM tris HCL, 1 mM EDTA, 150 mM NaCl, 1% Triton X-100, 1 mM EGTA, and 0.5 mL/mL of protease inhibitor (Bio Basic Inc., Markham, ON, Canada). The homogenate was then centrifuged at 10,000 rpm (Beckman Coulter, Indianapolis, IN, USA) for 35 min at 4 °C and the supernatant lysates were collected [76]. Protein content was assayed using the Bradford assay [77]. Measurement of β-amyloid peptide 1–42 concentration was measured using a rat ELISA Kit (WKEA Med Supplies Corp, Changchun, China) according to the manufacturer’s protocol. The color change was measured spectrophotometrically at a wavelength of 595 nm. The concentration of β-amyloid peptide (1–42) was calculated based on standards and expressed in pg/mg of total protein.

#### 4.6.8. Assessment of Acetyl Cholinesterase (AChE, EC 3.1.1.7) Activity

AChE activity was determined based on the method previously described by Ellman’s group [78]. Tissue lysate (250 μL) was added to 25 μL 5,5’-dithio-2-nitrobenzoic acid (0.82 mg/mL) and followed by addition of 25 μL of the enzyme substrate- acetylthiocholine (6.7 mg/mL). The absorbance change during 5 min intervals was measured at 405 nm immediately. The cholinesterase activity was calculated using the estimated molar extinction coefficient ε = 14,150 M^−1^ cm^−1^. The enzyme activity was finally expressed in μmol of acetylthiocholine hydrolyzed/min/mg tissue.

#### 4.6.9. Assessment of Glutamate Neurotransmitter Level

Cerebral cortex lysates in PBS (10 mg/100 μL PBS) were thawed for glutamate measurement using a rat glutamate ELISA kit (MyBioSource, San Diego, CA, USA) and a microplate reader (BioTek, Winooski, VT, USA) at 450 nm. Glutamate concentration was calculated using a professional standard curve, as described by the manufacturer.

#### 4.6.10. Determination of Serotonin Neurotransmitter

Cerebral cortex lysates in PBS (10 mg/100 μL PBS) were thawed from −20 °C for serotonin assessment using rat serotonin ELISA kit (MyBioSource) by a synergy 2 Multi-Mode Reader microplate (BioTek) at 450 nm. Serotonin levels were read within 15 min after adding the stop solution, and calculated using the standard curve described by the vendor.

#### 4.6.11. Determination of Dopamine Neurotransmitter

Cerebral cortex lysates in PBS (10 mg/100 μL PBS) were thawed from −20 °C for dopamine measurements using rat dopamine ELISA kit (MyBioSource, San Diego, CA, USA) by a synergy 2 Multi-Mode Reader microplate (BioTek) at 450 nm. Dopamine levels were measured within 15 min after the addition of a stop solution, and calculated using a professional standard curve provided by the manufacturer.

#### 4.6.12. Determination of Brain 8-OHdG Using HPLC

Isolation and hydrolysis of cerebral cortex DNA was performed as previously described [79]. The hydrolyzed mixture was centrifuged, and the supernatants were injected into the HPLC. The separation of 8-OHDG was performed with an LC/Agilent 1200 series HPLC apparatus (Conquer Scientific, San Diego, CA, USA) using UV detectors. For chromatographic separation, we used C18 reverse phase columns in series (Supelco, 5 pm, I.D. 0.46 × 25 cm). The eluting solution was H_2_O/CH_3_OH (85:15 *v*/*v*) with 50 mM KH_3_PO_4_, pH 5.5 at a flow rate of 0.68 mL/min. The UV detector was set at 245 nm. The resulting chromatogram identified the concentration from the sample as compared to that of the standard purchased from Sigma-Aldrich.

#### 4.6.13. Estimation of the Electrolytes

The electrolyte levels of the cerebral cortex homogenate were assessed spectrophotometrically. Sodium ion concentrations were estimated in an acid medium based on the principles described previously by Trinder [80]. The concentration of potassium ions was estimated according to the methods described by Sunderman and Sunderman [81]. Calcium ion concentrations were determined by the method of Grindler and King [82], which is based on the fact that calcium ions interact with methylthymol blue in an alkaline medium, a blue color that is in proportion to the calcium concentration.

### 4.7. Histopathological Assessment

Tissue specimens were dehydrated in a graded isopropanol series, cleared in xylene, immersed in Paraplast wax, and sectioned at 5-µm thickness using normal microtome. Cerebral cortex sections were mounted on coated slides (Thermo Scientific, Menzel-Gläser, Braunschweig, Germany) and consequently deparaffinized, then stained with hematoxylin and eosin (H&E) for histopathological manifestation.

### 4.8. Expression of Results and Statistical Significance

All data are presented as mean ± standard error of mean (SEM) of six replicates (*n* = 6). Data were analyzed using the GraphPad Prism V6.01 software package (San Diego, CA, USA). One-way analysis of variance (ANOVA) was used to test the significance of differences between treatments and the control as well. Pearson’s correlation was used to analyze the relationship between different variables. Statistical significance was set at *p* < 0.01 and *p* < 0.05.

## Figures and Tables

**Table 1:** Wave number assignments of FTIR spectra of ginger and propolis powder.

Ginger Powder		Propolis Powder	
Wave Number (cm^−1^)	Assignments	Wave Number (cm^−1^)	Assignments
3422.96	υ (O–H) cryst. water	3389.7	υ (O–H), υ (NH)
2923.38	υ_ass_ (CH2)	2924.13	υ_ass_ (CH2)
2377.44	----------	2377.55	-------------
2053.16	----------	2152.7	υ_ass_ (-C≡C-)
1641.47	δ (H_2_O), amide I (C=O)	2075.96	-------------
1458.21	Aromatic skeletal combined with C–H in plane deforming and stretching	1647.59	δ (H_2_O), amide I (C=O)
1374.64	Aromatic skeletal combined with C–H in plane deforming and stretching	1545.6	δ (HNH), (N–O), Q(C=O)
1245.25	υ (C–O–C), υ (C–F)	1425.68	υ (C–H) wagg., δ (-CH=CH-)
1158.76	υ (C–O–C), υ (C–F)	1373.73	Aromatic skeletal combined with C–H in plane deforming and stretching
1082.81	υ (C–O–C), υ (C–F)	1246.75	υ (C–O–C) or acid, υ (C–F)
1025.34	Amino acid, δ (C–H), υ (C–C)	1153.72	υ (C–O–C) or, υ (C–F)
850.75	υ (=C–H), δ (C–CL)	1023.08	υ (C–O–C) or, υ (C–F)
763.65	υ (C–H), δ (C–CL)	863.1	υ (=C–H)
703.4	υ (C–H), δ (C–CL)	768.52	υ (C–H), δ (C–CL)
577.29	δ (C–Br)	709.66	υ (C–H), δ (C–CL)
		573.5	δ (C–Br)

δ, rocking; υ, stretching; υ_ass_, asymmetric stretching; υ_s_, symmetric stretching; FTIR, Fourier transform infrared.

**Table 2:** Effect of ginger and propolis on calcium, sodium and potassium levels of brain tissues in MSG-treated rats.

Groups	Calcium (mmol/L)	Sodium (mmol/L)	Potassium (mmol/L)
Normal control (drinking H_2_O)	0.598 ± 0.048 ^b^	157.949 ± 1.8 ^b^	10.929 ± 0.51 ^b^
Monosodium glutamate (MSG)	0.941 ± 0.011 ^a,^*	192.268 ± 3.5 ^a,^*	6.790 ± 0.35 ^a,^*
MSG + Ginger	0.675 ± 0.063 ^b^	164.947 ± 4.1 ^b^	8.739 ± 0.44 ^a,b^
MSG + Propolis	0.594 ± 0.021 ^b^	164.161 ± 3.3 ^b^	9.112 ± 0.49 ^a,b^

Data are expressed as mean ± SEM (*n* = 6). In the columns, mean values with the same superscript letters are non-significant; otherwise, there is a significant difference. ^a^ Significantly different from normal control group at *p* ˂ 0.05 (*). ^b^ Significantly different from the MSG group at *p* ˂ 0.05 (*).

## Abbreviations

The following abbreviations are used in this manuscript:

AchE	Acetylcholinesterase
CAT	Catalase
CNS	Central nervous system
DPPH	α,α-Diphenyl-β-picrylhydrazyl
EGTA	Ethylene glycol-bis(β-aminoethyl ether)-*N*,*N*,*N*’,*N*’-tetraacetic acid
FTIR	Fourier transform infrared spectroscopy
GSH	Glutathione
GST	Glutathione S-transferase
FTIR	Fourier transform infrared
iNOS	Inducible nitric oxide synthase
MDA	Malondialdehyde
MSG	Monosodium glutamate
NO	Nitric oxide
8-OHdG	8-Hydroxy-2’-deoxyguanosine
ONOO^−^	Peroxinitrite radical
ROS	Reactive oxygen species
SOD	Superoxide dismutase
TBA	Thiobarbituric acid
TCA	Trichloro acetic acid
